# Co-occurrence of Dermatomyositis and Polycythemia Unveiling Rare *de Novo* Neuroendocrine Prostate Tumor

**DOI:** 10.3389/fonc.2018.00534

**Published:** 2018-11-20

**Authors:** Charalampos Papagoras, Stella Arelaki, Ioannis Botis, Ioannis Chrysafis, Stavros Giannopoulos, Panagiotis Skendros

**Affiliations:** ^1^First Department of Internal Medicine, University Hospital of Alexandroupolis, Democritus University of Thrace, Alexandroupolis, Greece; ^2^Department of Pathology, University Hospital of Alexandroupolis, Democritus University of Thrace, Alexandroupolis, Greece; ^3^Department of Radiology, University Hospital of Alexandroupolis, Democritus University of Thrace, Alexandroupolis, Greece; ^4^Department of Urology, University Hospital of Alexandroupolis, Democritus University of Thrace, Alexandroupolis, Greece

**Keywords:** prostate cancer, neuroendocrine neoplasia, dermatomyositis, polycythemia, erythrocytosis

## Abstract

We present a case of dermatomyositis together with polycythemia as initial manifestations of a particularly rare type of prostate cancer. A 69-year-old man was hospitalized for facial erythema and symptoms of fatigue. Physical evaluation, serum creatinine phosphokinase and electromyography were consistent with dermatomyositis. In parallel, the hemoglobin level was 18.5 g/dL, serum erythropoietin levels were low normal and no *JAK2* mutation was found. Given a strong suspicion of a paraneoplastic syndrome the patient underwent abdominal computed tomography revealing a prostate mass, enlarged iliac lymph nodes and a fracture of L1 due to metastasis. The unusual paraneoplastic manifestations prompted a more thorough immunohistologic examination of the needle biopsy specimen taken from the prostate, which led to the diagnosis of large cell neuroendocrine prostate carcinoma. It is a most rare type of prostate cancer, carrying a poor prognosis. To our knowledge, this is the first case in the literature associating a neuroendocrine cancer of the prostate with dermatomyositis.

## Introduction

The association between idiopathic inflammatory myopathies, particularly dermatomyositis, and cancer is a well-established observation. The most common type of cancer underlying dermatomyositis is adenocarcinoma, and the most commonly affected sites are the lung, ovary, breast, colon, uterus cervix, bladder, nasopharynx, esophagus, pancreas, and kidney (1). Although several other less frequent types of malignancy have been reported in patients with myositis, herein we report a case of an extremely rare type of prostate cancer manifesting with dermatomyositis and polycythemia.

## Background

**Figure 1:**
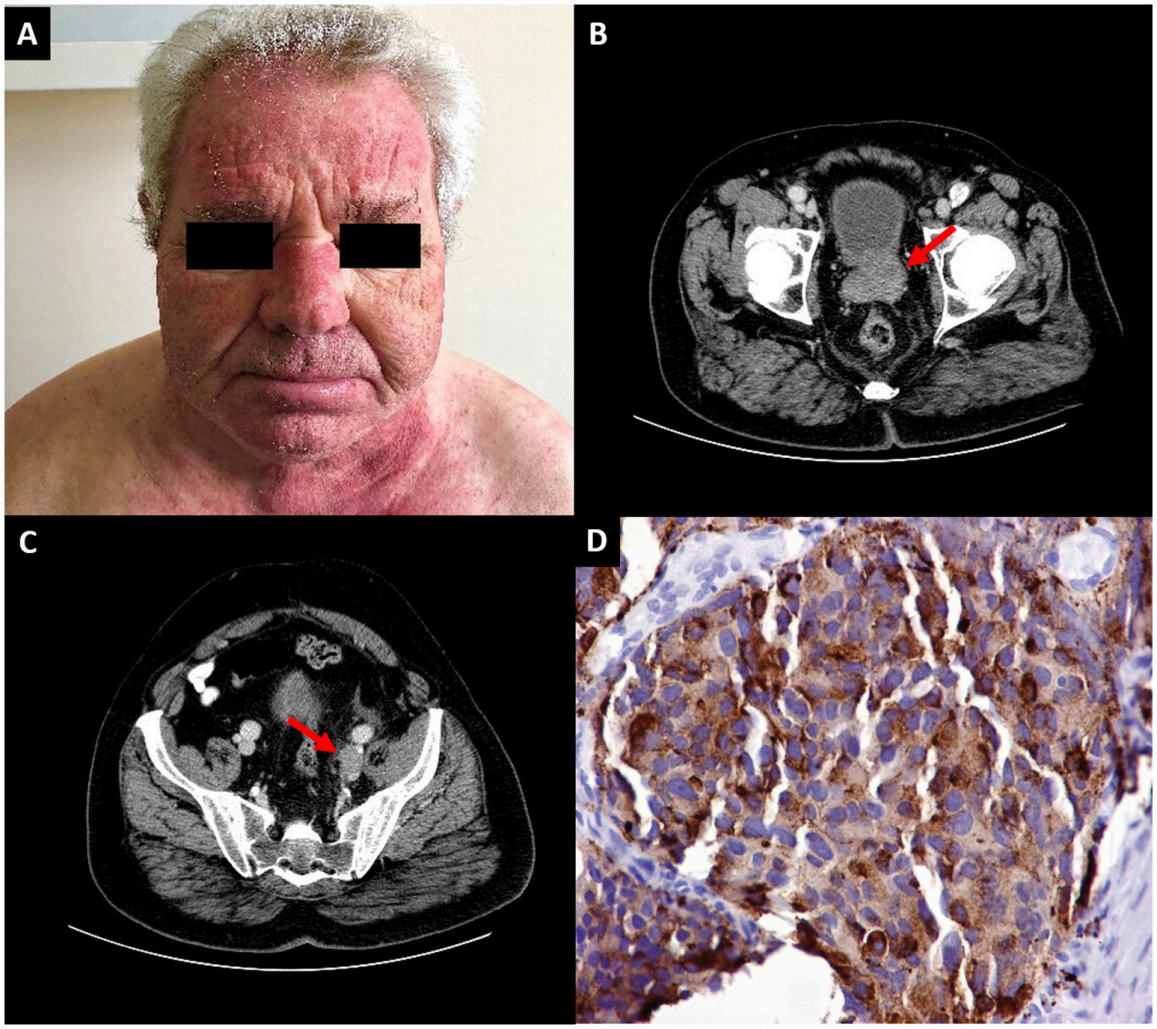
**(A)** Diffuse erythema of the face and the “V-neck sign,” suggestive of dermatomyositis. **(B)** Computed tomography of the abdomen revealing a prostate tumor (arrow) and **(C)** an enlarged left iliac lymph node (arrow) **(D)** Immunohistochemical section of a needle core biopsy from the prostate of the patient showing large cells staining positive for chromogranin A.

A 69-year-old man was admitted to the hospital for recurrent facial swelling and redness, accompanied by worsening fatigue during the previous 3 months. The episodes had initially been considered allergic and responded to short courses of glucocorticoids, only to recur a few weeks later. He had a history of arterial hypertension and an endovascular aortic aneurysm repair 6 years ago and his chronic medications included perindopril and low dose aspirin. He reported no significant urinary symptoms. Upon examination he had a reddish periorbital edema and an erythema of the face and the neck V-region (Figure 1A). Importantly, there was muscular weakness affecting the trunk and the proximal muscles of the extremities (grade 3 in a 0–5 scale). Apart from raised inflammatory markers (erythrocyte sedimentation rate 56 mm/h, C-reactive protein 6.1 mg/dL), serum creatinine phosphokinase was also elevated (1,819 U/L, normal 32–294). Electromyography of both proximal and distal muscles of the upper and lower limbs revealed scattered myopathic motor unit potentials, spontaneous fibrillations and increased insertional irritability, confirming the diagnosis of dermatomyositis.

In parallel, the patient had polycythemia (hematocrit 54.2%, hemoglobin 18.5 g/dL). Regarding possible causes, the patient's arterial pO_2_ was 80 mmHg on room air, serum erythropoietin level was low-normal (5 mU/mL, range 4.3–29) and a test for *JAK2* mutation was negative. The co-occurrence of late-onset dermatomyositis and polycythemia of undefined cause raised strong suspicions of an underlying malignant disease. A subsequent computed tomography scan of the abdomen revealed a large prostate tumor (Figure 1B), enlarged left iliac lymph nodes (Figure 1C) and a heterogeneous osteosclerosis of the left side of the L1 vertebral body with a collapse of its upper part and paravertebral fat edema, consistent with a metastasis at the L1 vertebra. The serum prostate specific antigen (PSA) was 11.49 ng/mL (normal 0–4). Remarkably, at this time point, symptoms and signs consistent with enlarged prostate were not yet apparent.

Needle core biopsy from the prostate revealed extensive sheets from large sized cells with prominent nucleoli, abundant cytoplasm and no prominent glandular differentiation. The tumor featured plenty of mitoses and apoptotic bodies. Immunohistochemical stains were strongly positive for neuroendocrine markers chromogranin A (Figure 1D) and synaptophysin and negative for the CD56 antigen. Interestingly, prostate markers such as PSA and prostatic specific acid phosphatase (PSAP) were positive, but only in a localized and weak pattern. The Ki-67 proliferative index was up to 70% of tumor cells. The histopathological report was of a large cell neuroendocrine prostate carcinoma (LCNPC). Therefore, the patient was diagnosed with metastatic LCNPC with paraneoplastic dermatomyositis and polycythemia and was referred to the Oncology department for treatment. However, the response to treatment was poor and he died 4 months after the diagnosis. Written informed consent was obtained from his wife for the publication of this case report.

## Discussion

We present a case of a very rare type of prostate cancer that manifested with symptoms of dermatomyositis and concomitant laboratory findings of erythrocytosis. The case is unique, because dermatomyositis or erythrocytosis in the course of large cell neuroendocrine prostate cancer have never been reported in the literature to our knowledge.

Idiopathic inflammatory myopathies are the rheumatologic diseases most strongly associated with cancer. Dermatomyositis seems to carry the highest relative risk compared to the general population, up to 5.5, followed by polymyositis with a relative risk 1.62 (2). Immune-mediated necrotizing myopathy, a recently described type of myositis, has also been linked to cancer, particularly in patients negative for antibodies against signal recognition particle (SRP) or positive against 3- hydroxy-3- methylglutaryl-coenzyme-A reductase (HMGCR) (3), although the latter are associated with statin-associated autoimmune myopathy as well (4). The risk of malignancy is usually highest the year preceding or following the myositis diagnosis and it seems to wane within 1–5 years post myositis diagnosis (1). Therefore, the most important issue is the recognition of prognostic factors to identify patients at risk, in order to screen for cancer and follow up appropriately. In a recent meta-analysis, risk factors for dermatomyositis-associated cancer were male sex, older age, cutaneous necrosis, elevated inflammatory markers and positivity for the anti-p155 antibody, which is thought to be directed against Transcription Intermediary Factor 1 γ (TIF-1γ). On the other hand, the presence of anti-Jo-1 and anti-Nuclear Extractable Antigen antibodies negatively associated with the risk of malignancy (5). Our patient had dermatomyositis with several of those risk factors (male, older age, elevated inflammatory markers), although we did not test for anti-p155/TIF-1γ antibodies. A possible mechanism underlying the paraneoplastic manifestations could be the production of antibodies against tumor antigens cross-reacting with muscle antigens (3, 6).

Erythrocytosis secondary to cancer is a recognized paraneoplastic manifestation of several types of cancer, such as hepatic or renal cancer (7). It may arise due to the ectopic production of erythropoietin or other erythropoiesis-inducing molecules from malignant cells (8). In a case report of a patient with colon adenocarcinoma and erythrocytosis, the colonic malignant cells expressed erythropoietin, although serum erythropoietin levels were also low normal, suggesting that the serum erythropoietin levels might be disproportionately high relative to the level of blood hemoglobin (7). As in our patient serum levels of erythropoietin were low normal as well, ectopic production of erythropoietin from the malignant neuroendocrine cells of the tumor cannot be ruled out, although immunohistochemistry for erythropoietin was not performed in the pathological specimen to confirm it.

Prostate cancer is the second most common malignancy in males and the second urological tumor associated with paraneoplastic manifestations after renal cell carcinoma (9). The vast majority are adenocarcinomas, while only 0.5% of tumors are classified as neuroendocrine (10). Among them, the occurrence of LCNPC is extremely rare, described only in case reports. The etiology of LCNPC is unknown. Most cases occur after androgen deprivation therapy for prostate adenocarcinoma (11). However, there have been reports of very few cases developing either *de novo* (11–14) or with advanced high-grade prostate adenocarcinoma (11, 15). The prognosis is poor with an average survival of < 1 year, as they are usually diagnosed in a metastatic stage (10).

The clinical manifestations, as well as the histopathologic features of a prostate neuroendocrine carcinoma may be similar to a high-grade prostatic adenocarcinoma. Moreover, neuroendocrine tumors are often characterized by inappropriate secretion of peptides, amines or other bioactive substances causing related syndromes. For instance, secretion of serotonin is the hallmark of the carcinoid syndrome, which is manifested with flushing of the skin, diarrhea, abdominal pain, right-sided heart-disease including right heart valve fibrosis and rarely myopathy, arthropathy and pigmentation (9). A great variety of other paraneoplastic signs and symptoms, such as hypercalcemia, syndrome of inappropriate antidiuretic hormone production, Cushing syndrome, Lambert-Eaton myasthenic syndrome, peripheral neuropathy or limbic encephalopathy, occur in association with genitourinary cancers, particularly small cell carcinomas, including those of the prostate (9, 16). However, such manifestations have not been reported with large cell neuroendocrine carcinoma of the prostate.

The key to diagnosis of this type of cancers is a neuroendocrine-orientated immunostaining, which, as in this case, should be prompted by the heterogeneous prostate enlargement and, particularly, the unusual symptoms which rarely occur in the common prostate adenocarcinoma (17). Serum PSA, a frequently used marker for prostate cancer screening of at-risk patients (including males over 50 years with myositis), may not be helpful, as there are several cases of neuroendocrine prostate cancer lacking significant PSA elevations despite the existence of a bulky tumor.

## Concluding remarks

In conclusion, we present an extremely rare case of a *de novo* large cell neuroendocrine prostate carcinoma which manifested with features of dermatomyositis and polycythemia, while urinary symptoms were absent. A high grade of suspicion for an underlying malignancy and the unusual systemic manifestations prompted a deeper scrutiny of the histopathological specimen revealing this rare type of cancer, which is treated through a different approach from classic prostate adenocarcinoma.

## Ethics statement

This report was in accordance with the rules of Ethics Review Board of the University Hospital of Alexandroupolis.

## Author contributions

CP treated the patient and drafted the manuscript. SA performed the histopathological tests and drafted the manuscript. IB and SG treated the patient and reviewed the manuscript. IC performed the imaging tests and reviewed the manuscript. PS treated the patient, reviewed the manuscript and supervised the work.

### Conflict of interest statement

The authors declare that the research was conducted in the absence of any commercial or financial relationships that could be construed as a potential conflict of interest.

## Abbreviations

HMGCR: 3- hydroxy-3- methylglutaryl-coenzyme-A reductase
LCNPC: Large cell neuroendocrine prostate carcinoma
PSA: Prostate specific antigen
PSAP: Prostatic specific acid phosphatase
SRP: signal recognition particle
TIF-1γ: Transcription Intermediary Factor 1 γ.
